# A Hybrid Approach to Short-Term Load Forecasting Aimed at Bad Data Detection in Secondary Substation Monitoring Equipment

**DOI:** 10.3390/s18113947

**Published:** 2018-11-14

**Authors:** Pedro Martín, Guillermo Moreno, Francisco Javier Rodríguez, José Antonio Jiménez, Ignacio Fernández

**Affiliations:** Department of Electronics, University of Alcalá, Alcalá de Henares, 28805 Madrid, Spain; martin@depeca.uah.es (P.M.); guillermo.morenob@uah.es (G.M.); jimenez@depeca.uah.es (J.A.J.); nacho.fernandez@uah.es (I.F.)

**Keywords:** measurement errors, singular spectrum analysis (SSA), artificial neural networks (ANN), bad data (BD) detection

## Abstract

Bad data as a result of measurement errors in secondary substation (SS) monitoring equipment is difficult to detect and negatively affects power system state estimation performance by both increasing the computational burden and jeopardizing the state estimation accuracy. In this paper a short-term load forecasting (STLF) hybrid strategy based on singular spectrum analysis (SSA) in combination with artificial neural networks (ANN), is presented. This STLF approach is aimed at detecting, identifying and eliminating and/or correcting such bad data before it is provided to the state estimator. This approach is developed to improve the accuracy of the load forecasts and it is tested against real power load data provided by electricity suppliers. Depending on the week considered, mean absolute percentage error (MAPE) values which range from 1.6% to 3.4% are achieved for STLF. Different systematic errors, such as gain and offset error levels and outliers, are successfully detected with a hit rate of 98%, and the corresponding measurements are corrected before they are sent to the control center for state estimation purposes.

## 1. Introduction

Power system state estimation (PSSE) has become an essential tool of energy and distribution management systems (EMS, DMS) aimed at controlling and planning of electric power grids [1,2]. With the advent of renewable energy along with the penetration of distributed energy resources (DERs), state estimation is assuming an expanded and prominent role. Determining factors, such as reliability and security, which have an influence upon service continuity, and economics, in terms of operating costs in power grids, greatly depend on the accuracy of the system state estimation, which in turn relies on the availability of reliable measurement data taken throughout the power system.

The power system state estimation is implemented by the control center (CC) which receives the measurement sets obtained from all the substations located throughout the network. These measurements range from bus voltages to bus real and reactive power injections, and branch reactive power flows in the power grid. Each substation is equipped with electronic instrumental transducers (EITs) which collect the analogue signals, process them and send the data to the CC.

The control center assumes, therefore, a crucial role in checking that the system satisfies the operational goals and ensures the power system reliability and security through the analysis of meter measurement data and power system models. However, the measurement data is not always accurate, on account of electromagnetic interferences, defective meters, noise in instruments and communications channels, errors in modeling pseudo measurements and false data injection attacks [1,3]. Such inaccurate measurements are commonly referred to as “bad data” (BD).

Many contributions to state estimation (SE) algorithms have been made in the past few decades [2,4,5,6,7,8]. Among them, weighted least squares (WLS) or least absolute value (LAV) estimators are commonly used in SE [4,5,8] Furthermore, the availability of historical databases allows the extraction of information necessary to produce measurement forecasts, which can be integrated into the SE process. This scheme is known as forecasting-aided state estimation (FASE) [6,9]. These methods are, however, significantly affected by bad measurements resulting in poor state estimates [2,6]. Consequently, a pre-estimation BD detection in measurements is an important part of the SE problem. Unfortunately, not all BD can be detected and eliminated by a pre-estimation process. Therefore, virtually all WLS-based estimators systematically implement post-estimation detection and identification methods with more advanced features [4].

In general, SE methods along with BD detection suffer from a main drawback, which questions the feasibility of their implementation in a real-time framework: the computational complexity, which is approximately proportional to the number of measurements used by the state estimator in the presence of BD. For instance, the computation of the residual covariance matrix in WLS algorithms, which requires the calculation of a subset of the elements in the inverse of the sparse gain matrix, is computationally intensive [8]. Furthermore, the processes of SE and BD detection need multiple state estimations, i.e., it is an iterative process, which further increases the computational time since a re-estimation of the system is needed after every BD elimination.

To overcome this drawback, the amount of BD provided to the state estimator should be reduced by implementing a BD pre-filtering stage at a decentralized level in the substations. The proposed approach has all the advantages of decentralized methods: (1) scalability which is increased by reducing the communication overhead; (2) robustness to changes in the network configuration; (3) and computation and bandwidth overhead reduction at the control center level. More importantly, this decentralized approach eases the computational burden in the control center. Whereas BD post-estimation has received a lot of attention in literature in the past few years, BD pre-filtering lacks systematic procedures or new techniques at a decentralized level. The approach presented in this paper takes advantage of the computational resources, which are being installed in Secondary Substations in order to extend the real-time monitoring to Low Voltage (LV) grids. This is one of the main contributions of this paper.

This paper presents a strategy to filter the quantity of BD provided to the SE algorithms thereby significantly reducing the number of iterations required for the SE and BD detection and elimination. In particular, the strategy developed is aimed at detecting and identifying permanent meter failure, namely offset and gain errors, and meter malfunction in the measurement equipment (EITs), installed in secondary substations. The technique is based on short-term load forecasting (STLF), which in turn relies on a hybrid singular spectrum analysis—artificial neural network (SSA-ANN) approach for improved forecasting accuracy. Generally, in secondary substations, STLF has not been commonly used as an effective tool for error detection. It should be noted that at SS level, erroneous data is prevalent (up to 25% inaccurate measurements) which seriously jeopardizes the SE accuracy and forecasting [10].

In the literature, there are many examples of using hybrid approaches based on SSA for forecasting in many different fields. In [11] SSA is used for eliminating the noisy component of the time series and a local linear neuro-fuzzy model (LLNF) to forecast several well-known time series with different structures and characteristics. An integrated model based on ANN and SSA is presented in [12] and is applied to forecast short-term wind speed. Recently, this model has also been used in [13,14] for precipitation forecasting. In [15] authors study the importance of hybrid models for forecasting of hydrological data. The hybrid SSA-ANN model used in this paper differs from previous works in several ways: firstly, the grouping stage is automatized; secondly, it considers the residual as an important piece of information for the forecasting process; finally forecasting results are used for BD detection.

The remainder of this paper is organized as follows. In Section 2 a global description of the system and the strategies developed for the BD detection are described. Section 3 analyzes the STLF framework, and the results achieved are shown in Section 4. Finally, some conclusions are drawn in Section 5.

## 2. System Description

Secondary substations (SSs) are being provided with more intelligence in order to get a more accurate and realistic view on LV grids and to filter and analyze data before it is sent to the control center [16]. Consequently, SSs can play an important role in this decentralized architecture, allowing the data flow to be optimized, thereby reducing the data that reaches the control center for analysis and decision-making.

**Figure 1:**
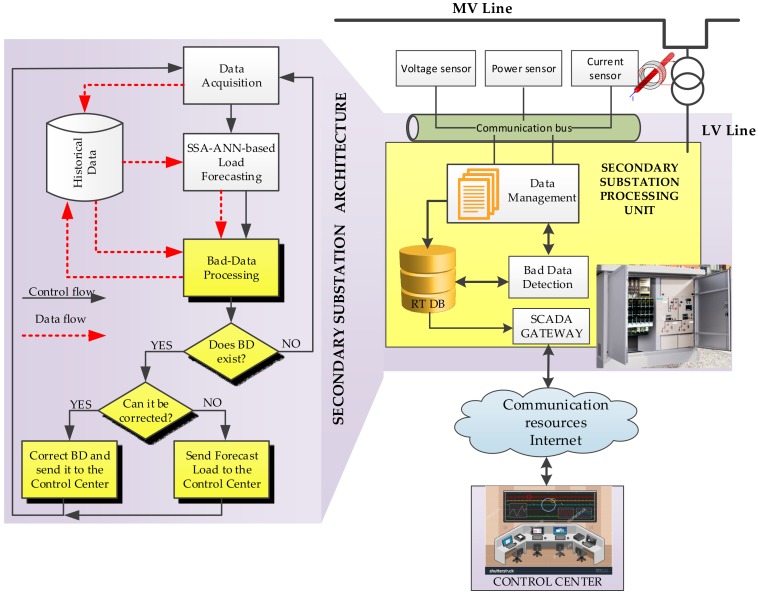
General view of the system implemented in the SS.

With this in mind, Figure 1 shows a general view of the system presented in this paper and a flowchart of the algorithm implemented based on an iterative process. The main software components are represented in the substation processing unit. The data management module collects substation measurement data and stores it in the real time data base (RT DB). This data management module is, therefore, connected to the LV side by current transformers (CTs) and voltage transformers (VTs), which measure currents and voltages respectively. Furthermore, measurement units provide active and reactive power every ten minutes. For the case study, hourly average active power is considered. All the measurements are stored in the local data base, although they can be read and stored remotely in the control center database. 

The bad data detection module implements the hybrid SSA-ANN-based algorithm for the detection, identification, and correction of the BD. This algorithm is depicted in the flowchart in Figure 1. By using the proposed algorithm, BD is both detected and identified in the following step (bad data processing). Gain and offset errors in the measurement equipment are detected, quantified and used to correct, if possible, the erroneous measurements before they are sent to the control center. In the event of erroneous measurements, which do not fall into the patterns for the gain and the offset errors, forecast measurements are sent instead.

## 3. Short Term Load Forecasting Framework

**Figure 2:**
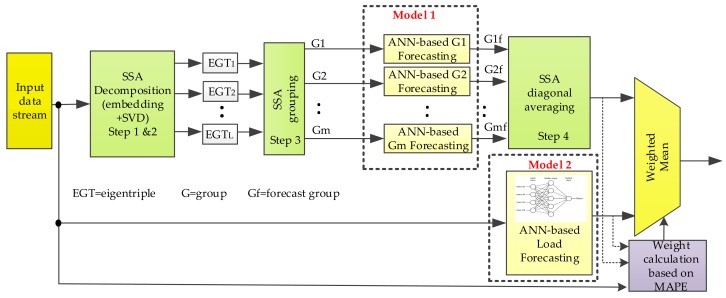
Hybrid forecasting framework.

In this section, the hybrid approach for STLF based on singular spectrum analysis (SSA) and artificial neural networks (ANN) is explained. In Figure 2 a block diagram of the prediction is shown in outline. The algorithm proposed in this paper consists of three different steps: (1) data preprocessing based on a SSA decomposition and a grouping stage to obtain the independent components of the time series (Model 1), namely trend, periodicities and residual. These components are then individually forecasted before the reconstruction step. By using SSA the forecasting process is boosted, since the extracted components show less complexity than the original time series, thereby facilitating the ANN learning process; (2) ANN-based STLF step in which the whole time series is predicted based on ANNs (Model 2); and (3) finally, by using mean absolute percentage error (MAPE), the weighted arithmetic mean of both forecasting datasets is calculated. 

From a practical point of view, the process of forecasting works as follows. The input data stream (Figure 2) is provided by the data acquisition module (Figure 1) and the database on which the historical data is stored. This dataset is simultaneously fed to the SSA-ANN-based forecasting model (Model 1) and ANN-based forecasting model (Model 2). Regarding Model 1, by using SSA the dataset is decomposed in several additive components in the shape of groups of eigentriples which are individually forecasted. Then a 24-h prediction of the load is obtained through a SSA-based reconstruction stage. As far as Model 2 is concerned, the 24-h forecast is carried out by using an ANN-based strategy. Finally, the accuracy of both forecasts is assessed by using MAPE and their weighted mean constitutes the final prediction. It is, therefore, a combined strategy which chooses the more accurate prediction of the two models mentioned before, by giving more priority to that prediction with less MAPE. The weights are updated on a daily basis so as to compensate for any time-dependent variation.

In principle, ANNs should be able to approximate and generalize any nonlinear function to any degree of accuracy. The load dataset is a time series which displays trend and different levels of seasonality (daily, weekly and annual), which makes it nonstationary. The nonstationary aspect of many time series is a significant factor which hinders the ANN prediction performance. Therefore, it seems reasonable to remove from the load series the somewhat deterministic information, enhancing the ANN learning ability for modelling the important features. However, in STLF, seasonal patterns can interact with exogenous variables, mainly meteorological variables, and clearly influence the load. Therefore, short-term load forecasting strategies should take seasonal patterns into consideration to capture this correlation, with the exception of the annual seasonality, since STLF timeframes are substantially shorter in length than the annual cycle.

As far as the trend is concerned, ANN-based forecasting of trend time series is not straightforward on account of the bounded feature of the activation function used for the neurons of the hidden layer. This fact can have undesired effects on the dataset normalization i.e., values that are out of the range used for normalization. It is important to note that in STLF a trend is a valuable indicator of a systematic drift in the data caused by, for instance, a sensor drift. Hence, removing the trends from the data may be detrimental, since trends are useful in detecting systematic errors in measurements. In order to deal with this issue, expected values of trend caused by systematic errors are considered when normalizing. In this work the inputs to the ANN have been normalized to [−1,1].

### 3.1. Introduction to Singular Spectrum Analysis

Basic SSA decomposes a time series $Y_{T}=\left(y_{1},\ldots ,y_{T}\right)$ of length T into several components and reconstructs the series by eliminating the noise, i.e., the high frequency components of the noisy power load, provided that the signal can be separated from the noise. SSA consists of two main stages namely, decomposition and reconstruction, which, in turn, include two separate steps. The first step in the SSA decomposition is called embedding and consists in defining the trajectory matrix, $X=\left[X_{1},\ldots ,X_{K}\right]$ where $X_{i}=\left(y_{i},\ldots ,y_{i+L-1}\right)$, the number of columns K=T−L+1 and the window length L≤T/2. The trajectory matrix X is based on L lagged copies of the time series and can be written as follows:

(1) $$\;X=\left[X_{1},\ldots ,{\;X}_{K}\right]=\left(\begin{array}{ccc} \begin{array}{cc} y_{1} & y_{2} \end{array} & \cdots & y_{K}\\ \vdots & \ddots & \vdots \\ \begin{array}{cc} y_{L} & y_{L+1} \end{array} & \cdots & y_{T} \end{array}\right)$$

The value of the window length L depends upon the time series features. For instance, when the time series includes an integer-period component, L should be defined proportional to that period. The aim is to select a window length that produces separable and independent components to capture the trend and oscillations in the time series. Hence it is important that each L lagged copy of the time series incorporates an essential part of the behavior of the initial series [17,18]. The second step consists in computing the matrix $S=X\;\cdot {\;X}_{T}$ and determining the eigenvalues $\lambda_{i}$ being  i=1,…,L taken in the decreasing order of magnitude $\left(\lambda_{1}\geq \lambda_{2}\geq \ldots \geq \lambda_{d}\geq 0\right)$ where $d=\max\left\{i\;:{\;\lambda }_{i}>0\right\}$ and the corresponding eigenvectors $U_{i}=\left(U_{1},{\;U}_{2},\ldots ,U_{d}\right)$ of the matrix S by applying singular value decomposition (SVD). The trajectory matrix X can then be expressed as the sum of d rank one elementary matrices $X=X_{1}\;+\;\ldots +{\;X}_{d}$ where d is the number of non-zero eigenvalues of the matrix S as stated above. The elementary matrices can be expressed by the triples:

(2) $$X_{i}=\sqrt{\lambda_{i}}U_{i}V_{i}^{T}$$

where $V_{i}=\left(X^{T}\cdot {\;U}_{i}\right)/\sqrt{\lambda_{i}}$, (i=1,…,d) are the factor vectors, $\lambda_{i}$ are the eigenvalues, $\sqrt{\lambda_{i}}$ are the singular values of X and the set $\left\{\sqrt{\lambda_{i}}\right\}$ is called the spectrum of X. The collection ($\sqrt{\lambda_{i}},{\;U}_{i},V_{i}$) is called ith eigentriple of the matrix X. Therefore, X can be expressed as follows:

(3) $$\;X=X_{1}+X_{2}+\ldots +X_{d}=\sqrt{\lambda_{1}}U_{1}V_{1}^{T}+\sqrt{\lambda_{2}}U_{2}V_{2}^{T}+\ldots +\sqrt{\lambda_{d}}U_{d}V_{d}^{T}$$

The plot of the singular values in decreasing order is called the singular spectrum and can be used to calculate the index r(r<d). Index r indicates the number of components corresponding to the largest eigenvalues of X that allows the signal from noise to be separated. In the simplest way, the grouping step consists in approximating X by the sum of the first r elementary matrices, and it can be expressed as:

(4) $$\;X\approx X_{1}+X_{2}+\cdots +X_{r}\;$$

Finally, the fourth step deals with the reconstruction of the one-dimensional series. By taking the average of the diagonals of each elementary matrix, elementary time series, which represent the different components of the behavior of the original time series, can be obtained.

**Figure 3:**
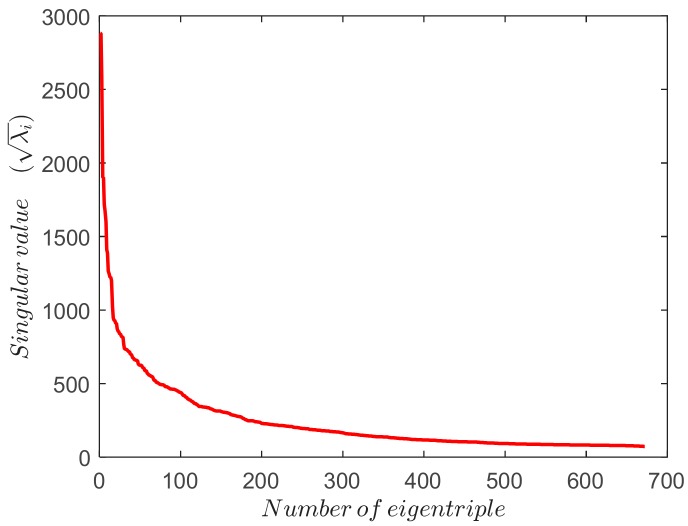
Singular spectrum.

The work presented in this paper introduces some improvements in order to automatize the grouping step of the algorithm so it can be used independently of the data being considered. To achieve this purpose, the grouping phase, based on some quality features, automatically selects those eigentriples that represent the trend and oscillations in the signal. Firstly, an initial window length L suitable for extracting the trend and most periodicities is selected. A value of L=24×7×4 (four weeks window length) works well as the main frequencies are dividers of this value or are close to it. In the second step, the number of eigentriples to represent has to be determined. The importance of each eigentriple is directly proportional to the amplitude of its eigenvalue, so it is possible from the singular spectrum (Figure 3) to select those eigentriples that contain the most information in the signal, i.e., the most variance explained. The remaining eigentriples constitute the residual, which still contains signal information as strong separability is not always possible. The absence of strong separability is not a problem since a well-trained ANN can extract the signal pattern from the noise in the residual.

**Figure 4:**
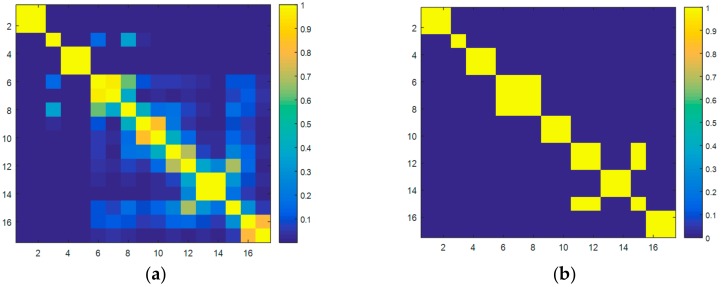
Correlation matrix. (**a**) Eigentriples selected. (**b**) Groups selected.

The eigentriples that carry the most variance explained are then grouped in the third phase of the SSA process. To do so, the weighted correlation of the eigentriples (Figure 4a) is used to cluster them when a percentage of correlation is accomplished. After several simulations, a minimum correlation of 0.6 has been selected to group the eigentriples (Figure 4b).

### 3.2. SSA-ANN-Based Load Forecasting Strategy

Both forecasting models (Model 1 and Model 2 in Figure 2) are based on neural networks on account of their approximation ability for non-linear mapping and generalization. Depending on the model considered, the process of forecasting s individually applied to each group of eigentriples from the SSA decomposition step and the residual (Model 1), or to the whole time series (Model 2). 

In this paper the ANN topology is based on a nonlinear autoregressive with exogenous inputs (NARX) model where the load is explained by the historical values of the load i.e., lagged observations, the temperature and the day of the week and month, which are crucial for the ANN to keep track of the seasonal components and weather fluctuations [19]. The ANN has three layers: (1) an input layer, whose inputs depend on the data to be forecast; (2) a hidden layer with a number of neurons, which depends on the complexity of the function to be learned and the training set used; and (3) a 24-neuron output layer since a static approach is implemented, which forecasts the 24-h load simultaneously. The transfer functions for the neurons of the hidden and output layers are log-sigmoid and linear respectively.

Regarding the input layer, the input data depends on the model. Whereas in Model 1, the input data set consists of different samples from the corresponding group of eigentriples, in Model 2, the input data set is based on the whole time series. In both cases the data structure consists of: (1) hourly load of the previous day; (2) hourly load of the previous week same day; (3) hourly load taken the same day, two weeks before the day to be forecasted; (4) hourly dry-bulb temperature of the same day; and (5) the weekday and the month, expressed by cosine functions. As for the hidden layer, in Model 1, all the ANNs used for forecasting the groups of eigentriples have 8 neurons in the hidden layer, which provide the best prediction results. As far as the residual is concerned, a 20-neuron hidden layer offers the best performance as they are more difficult to predict. In model 2, 20. neurons are used as well.

### 3.3. Bad Data Processing. Error Correction/Elimination

The next step in the algorithm presented in this paper consists in processing the BD due to offset and gain errors and measurements which are very large or very small. Offset and gain errors can be identified and corrected and/or eliminated before they are sent to the control center. However, complete elimination of these errors requires properly calibrated and maintained measuring equipment. Measurements which are very large or very small are commonly known as outliers, which must be removed from the data base and replaced by the forecast data in order to avoid missing data. An outlier detection method based on principal component analysis (PCA) has been used in this work [20,21].

**Figure 5:**
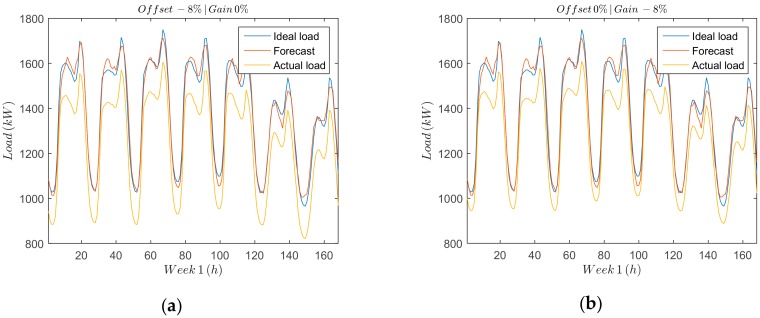
Errors to be detected. (**a**) Offset error. (**b**) Gain error.

Offset and gain errors (Figure 5a,b) can be easily detected and identified due to their systematic effects in the measurement data. It is important to characterize both errors so that the remaining sources of deviation of the measured data from the forecasting load can be attributed to random effects. Offset errors are additive and can be represented by a positive or negative term in the measured data.

Figure 5a shows the load measured deviation due to the offset error, which is compared against the full scale. Gain errors, on the other hand, are multiplicative and affect all the measurements proportionally. Figure 5b shows a comparison between the measured, the forecast and the ideal load, over a period of seven days for an offset error Figure 5a and a gain error Figure 5b of −8%.

The worst-case scenario is that both errors can occur together and with different signs. Considering that gain and offset errors are uncorrelated, they can be described as follows:

(5) $$\;m_{\mathrm{lp}}\left(t\right)=\left(i_{\mathrm{lp}}\left(t\right)\cdot \alpha \right)+\beta$$

where $m_{\mathrm{lp}}$ represents the measured load profile, $i_{\mathrm{lp}}$. is the ideal measurement load profile, i.e., when no errors occur, and α and β represent the gain and offset errors, respectively.

By using the strategy presented previously, the forecast load profile $f_{\mathrm{lp}}\left(t\right)$ can be determined. Since the process of forecasting introduces a random error $e_{f}\left(t\right)$, Equation (5) can be rewritten as:

(6) $$\;m_{\mathrm{lp}}\left(t\right)=\left(f_{\mathrm{lp}}\left(t\right)+e_{f}\left(t\right)\cdot \alpha \right)+\beta =f_{\mathrm{lp}}\left(t\right)\cdot \alpha +e_{f}\left(t\right)\cdot \alpha +\beta \;$$

Needless to say, the better the load prediction, the smaller the error introduced. However, this error can be negligible as it is a random error with mean 0. Therefore, by using different averaging processes, Equation (6) can be reformulated as:

(7) $$\;\bar{m_{\mathrm{lp}}}\left(t\right)=\bar{f_{\mathrm{lp}}}\left(t\right)\cdot \alpha +\beta \;$$

Differentiating both terms in Equation (7) leads to:

(8) $$\frac{\Delta \bar{m_{\mathrm{lp}}}\left(t\right)}{\Delta t}=\frac{\Delta \left(\bar{f_{\mathrm{lp}}}\left(t\right)\cdot \alpha +\beta \right)}{\Delta t}=\frac{\Delta \bar{f_{\mathrm{lp}}}\left(t\right)}{\Delta t}\cdot \alpha$$

then, the following equation can be obtained by solving for α:

(9) $$\alpha =\frac{\Delta \bar{m_{\mathrm{lp}}}\left(t\right)/\Delta t}{\Delta \bar{f_{\mathrm{lp}}}\left(t\right)/\Delta t}=\frac{\Delta \bar{m_{\mathrm{lp}}}\left(t\right)}{\Delta \bar{f_{\mathrm{lp}}}\left(t\right)}$$

Finally, substituting the gain error into Equation (7), and solving for β leads to:

(10) $$\beta =\bar{m_{\mathrm{lp}}}\left(t\right)-\bar{f_{\mathrm{lp}}}\left(t\right)\cdot \alpha \;$$

The proposed system is able to estimate the value of gain and offset errors independently. Erroneous measurements can be corrected if they follow the offset and gain pattern, i.e., if the deviation between the $m_{\mathrm{lp}}$ and $f_{\mathrm{lp}}$ can be expressed in terms of α and/or β and, therefore, a correction can be applied to compensate for the effect. Outliers, on the other hand, are replaced by forecasted values. The same applies for missing data.

### 3.4. Load Forecasting Accuracy Assessment: Weight Calculation

**Figure 6:**
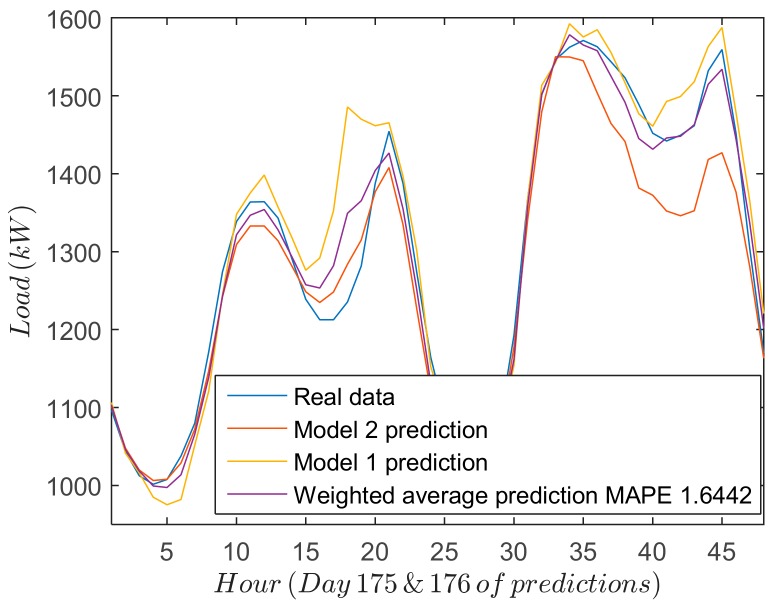
Forecasted data for two different consecutive days. First 24 h when Model 1 offers a bad prediction and last 24 h when the opposite occurs.

The performance of the different forecasting strategies is shown in Figure 6, which includes for two days: (1) real measurements; (2) forecasted results for both models; and (3) final results when the weighted average is applied. From the results yielded by both SSA-ANN (Model 1) and ANN (Model 2) approaches, some conclusions can be drawn. Firstly, the most significant deviations of the forecasting values from the real measurements occur at the turning points of the forecast load curve. Secondly, in some cases Model 1 offers a good prediction (last 24 h of Figure 6) whereas in others, Model 2 works better (first 24 h of Figure 6). Consequently, a combination of both models can offer a good global prediction.

The mean absolute percentage error (MAPE) is chosen to assess the forecast accuracy is defined as follows:

(11) $$\;\mathrm{MAPE}=\frac{1}{T}\cdot \overset{T}{\underset{t=1}{\sum }}\left|\frac{P_{\mathrm{tm}}-P_{\mathrm{tf}}}{P_{\mathrm{tm}}}\right|\cdot 100\;\left(\%\right)$$

where $P_{\mathrm{tm}}$ and $P_{\mathrm{tf}}$ denote the measured and forecast power at instant t, respectively, and T is the length of the horizon considered in the prediction, i.e., 24 h in this work.

The forecast time series for the power consumption can be written as:

(12) $$\;P_{\mathrm{tf}}=w_{f1}\cdot P_{\mathrm{tf}1}+w_{f2}\cdot P_{\mathrm{tf}2}$$

where $P_{\mathrm{tf}1}$ and $P_{\mathrm{tf}2}$ denote the forecast consumption for the Model 1 and Model 2 respectively and $w_{f1}$ and $w_{f2}$ are the corresponding weights that define the relative influence of the forecast power in the final prediction.

The weights are calculated by means of the individual MAPEs, as follows:

(13) $$w_{f1}=\frac{{\mathrm{MAPE}}_{2}}{{\mathrm{MAPE}}_{1}+{\mathrm{MAPE}}_{2}}{\;w}_{f2}=\frac{{\mathrm{MAPE}}_{1}}{{\mathrm{MAPE}}_{2}+{\mathrm{MAPE}}_{1}}$$

where ${\mathrm{MAPE}}_{1}$ and ${\mathrm{MAPE}}_{2}$ represent the forecasting error for the Model 1 and Model 2, respectively.

Mean errors may differ as a function of the day of the week, especially between working days and weekends. With the aim of averaging out these differences and updating the weights on a daily basis, the average MAPE for T forecast values and N days is considered, which can be formulated as:

(14) $${\mathrm{MAPE}}_{j}=\frac{1}{N}\cdot \overset{N}{\underset{n=1}{\sum }}\left(\frac{1}{T}\cdot \overset{T}{\underset{t=1}{\sum }}\left|\frac{P_{\mathrm{tmj}}-P_{\mathrm{tfj}}}{P_{\mathrm{tmj}}}\right|\cdot 100\right)\left(\%\right)$$

for j=[1,2], T=24 and N=7.

**Figure 7:**
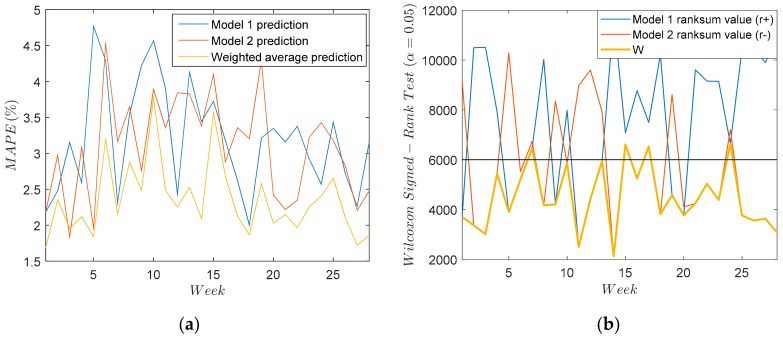
Model 1 and Model 2 comparison. (**a**) MAPE weekly prediction for both models and weighted average. (**b**) Weekly Wilcoxon signed-rank test under α=0.05.

In Figure 7a weekly MAPE is shown for every model. It can be observed that sometimes the first model shows better performance than the second and vice versa. However, the weighted average prediction always shows the best performance. As a result, the weighted average prediction will be used to detect gain and offset errors. In order to demonstrate the difference in forecasting accuracy between both models, the weekly Wilcoxon signed-rank test is carried out and its results are shown in Figure 7b. The Wilcoxon signed-rank test [22] is used to compare the significant differences in terms of central tendency between two equal-length data sets: the errors of the forecast strategies being compared. The statistic W of the Wilcoxon signed-rank test is shown as Equation (15):

(15) $$\;W=\min\left\{r^{+},r^{-}\right\}$$

where $r^{+}$ represents the sum of ranks in which the first model is larger than the second one and $r^{-}$ represents the sum of ranks when the opposite occurs. If W meets the criterion of the Wilcoxon distribution under N degrees of freedom, then, the null hypothesis of equal performance of these two compared models cannot be accepted [23,24].

Figure 7b shows the values of $r^{+}$ and $r^{-}$ for both models, and the value of W. If the value of W is above the black line depicted in the figure, the test indicates that the null hypothesis under α = 0.05 should be accepted, showing that both models are similar enough. As can be seen from the figure, the value of W is usually under the black line, this value corresponding indistinctly with Model 1 and in other cases with Model 2, so it can be assumed that the performance of both models are complementary and the weighted average prediction provides better results.

In the field of STLF many works have been published and many types of strategies have been used showing the good performance of the techniques proposed in the papers. In this regard, for comparison purposes, it is complicated to reproduce the experiments presented in other works because either, the data set is not available or the explanatory variables are different or simply because the experimental setup has not been described in detail. This clearly does not facilitate comparison. Another challenging issue is the lack of standardized benchmarks: there is no a widely accepted model and dataset for benchmarking in STLF. Finally, although many papers use MAPE for quality assessment, there is no uniformity and it can be used for different time scales and horizons. Nevertheless, in the case study presented in this paper, two benchmark models can be used in order to assess the quality of the predictions, namely: (i) Seasonal ARIMA model and (ii) SSA-based model. Both models can be used for benchmarking purposes since they are accurate enough to capture the prominent features of the predictor variables.

The MAPE, the normalized root mean square error (NRMSE) and their non-normalized expressions are used to compare results, and are defined by the following Equations (16)–(19):

(16) $$\;\mathrm{MAPE}=\frac{1}{T}\cdot \overset{T}{\underset{t=1}{\sum }}\left|\frac{P_{\mathrm{tm}}-P_{\mathrm{tf}}}{P_{\mathrm{tm}}}\right|\cdot 100\;\left(\%\right)$$

(17) $$\mathrm{MAE}=\frac{1}{T}\cdot \overset{T}{\underset{t=1}{\sum }}\left|P_{\mathrm{tm}}-P_{\mathrm{tf}}\right|\;\left(\mathrm{kW}\right)$$

(18) $$\mathrm{NRMSE}=\sqrt{\frac{1}{T}\cdot \overset{T}{\underset{t=1}{\sum }}{\left(\frac{P_{\mathrm{tm}}-P_{\mathrm{tf}}}{P_{\mathrm{tm}}}\right)}^{2}}\cdot 100\;\left(\%\right)$$

(19) $$\mathrm{RMSE}=\sqrt{\frac{1}{T}\cdot \overset{T}{\underset{t=1}{\sum }}{\left(P_{\mathrm{tm}}-P_{\mathrm{tf}}\right)}^{2}}\left(\mathrm{kW}\right)$$

where and $P_{\mathrm{tf}}$ denote the measured and forecast power at instant t, respectively, and T is the number of forecast days, i.e., 200 days in this work, considering a 24-h horizon.

The error indicators are shown in Table 1. It can be seen that the weighted average model gives the best performance.

## 4. Experimental Results: Error Detection.

The system described in this paper has been tested against real data sets obtained from different SSs located in the Community of Madrid. This data contains hourly recorded information from several SSs in the period ranging from 1 June 2013 to 31 December 2016.

The data set is split into three groups: (1) the training subset (70%), which is used to train the ANNs; (2) the validation subset (15%) used to ensure that the ANN is generalizing and to prevent overfitting; and (3) the test subset employed for a completely independent test of network generalization. Different values of offset and gain are inserted in the data set and are successfully detected.

Regarding gain and offset error detection, an approach to error analysis, similar to that introduced in [25], has been implemented. The equipment involved in the data acquisition, mainly current transformers and measurement equipment, introduces an inherent measurement error, the value of which has been quantified. The transformers account for an error not higher than ±1% [26,27] and the measurement equipment can introduce an error that ranges from ±0.5% to ±1% depending on the manufacturer [28]. Therefore, under normal operating conditions, an average value ±2% can be considered a conservative estimate for the maximum inherent error introduced by the measurement equipment. With this in mind and to avoid detection of false gain and offset errors, a minimum threshold of ±4% has been considered.

In order to obtain an average error value, the measured error estimation is filtered by using a recursive least square algorithm (RLS). A prediction time ($T_{p}$), which, in this context corresponds with the period of time considered to estimate the current gain and offset errors, must be defined. Needless to say, the longer the prediction time, the more precise the estimate. However, a $T_{p}$ which is too long can undermine the algorithm’s effectiveness, since an early error detection is to be desired. A $T_{p}$ of 7 days has therefore been defined considering that in measurement equipment, gain and offset errors evolve slowly in time.

In order to verify the accuracy of the detection of the normalized error, defined as the combination of the offset and gain errors, Equation (20) is used:

(20) $$\;\epsilon_{\mathrm{norm}}=\sqrt{\frac{{\left(\epsilon_{\alpha }-\alpha \right)}^{2}+{\left(\epsilon_{\beta }-\beta \right)}^{2}}{\epsilon_{\alpha }{}^{2}+\epsilon_{\beta }{}^{2}}\;}$$

where α and β represent the detected errors and $\epsilon_{\alpha }$ and $\epsilon_{\beta }$ are the injected errors for the gain and offset, respectively. Likewise, Equation (21) defines a threshold above which an alarm warning is generated. Taking into account the previously mentioned inherent error introduced by the measurement equipment, the threshold is set to 4%:

(21) $$\delta =\sqrt{\alpha^{2}+\beta^{2}\;}$$

By using Equation (21) and setting different values for $\epsilon_{\alpha }$ and $\epsilon_{\beta }$, ranging from ±1% to ±5%, several scenarios are considered (Table 2): (1) when both $\sqrt{\epsilon_{\alpha }{}^{2}+\epsilon_{\beta }{}^{2}}$ and δ are greater than 4%, this is regarded as a true positive (TP), which means that the combined error has been successfully detected, otherwise, i.e., when δ≤4%, it is a false negative (FN); and (2) when both $\sqrt{\epsilon_{\alpha }{}^{2}+\epsilon_{\beta }{}^{2}\;}$ and δ are less than or equal to 4%, this is regarded as a true negative (TN), otherwise, i.e., when δ>4%, it is a false positive (FP).

**Figure 8:**
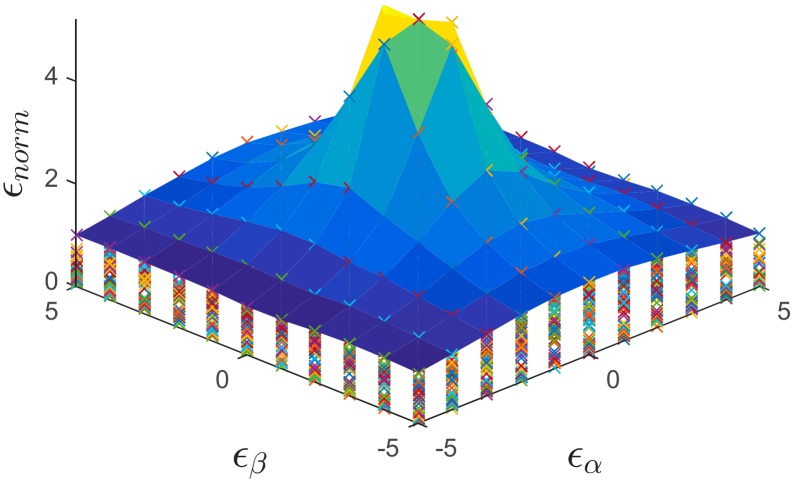
Normalized detected error ($\epsilon_{\mathrm{norm}}$) as a function of the injected error for the weeks under study.

**Figure 9:**
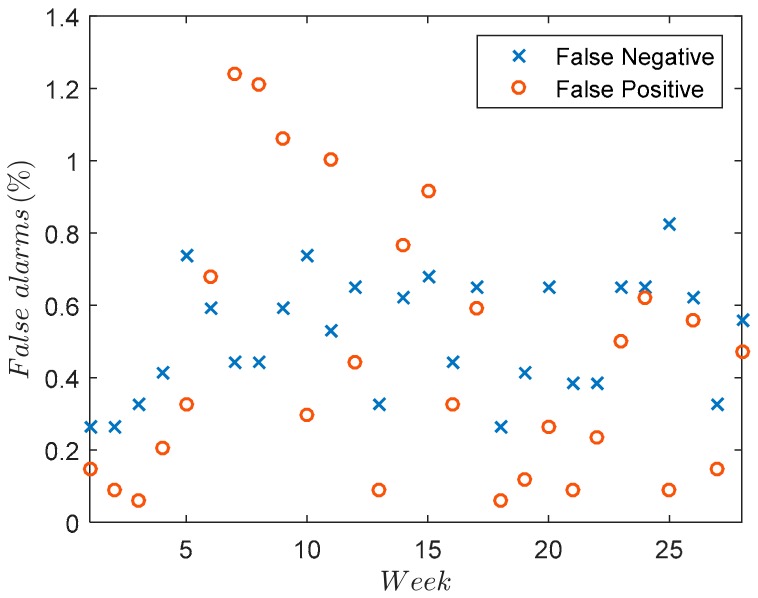
False alarms as a function of the week.

Table 3 depicts several values of $\epsilon_{\mathrm{norm}}$, which have been calculated for a random week. Figure 8, on the other hand, shows a more general case in which all the weeks for the error detection are considered. In this figure the envelope represents the worst-case scenario, i.e., the worst week regarding the detection errors for the offset and gain. It can be noticed from Figure 8, that the normalized error follows a zero-centered bi-dimensional normal distribution. Figure 9 shows, represented as a percentage, the false positives (FP) and false negatives (FN) for each week, taking into account the criterion explained in Table 2 and the values used in Table 3.

It can be concluded, therefore, that the algorithm accuracy does not greatly depend upon the existing offset and gain. However, this accuracy does depend upon the performance of the load forecasting algorithm.

## 5. Conclusions

Power system state estimation is a crucial functionality which ensures reliability and security of power grid operation. Measurements provided by secondary substations are important since LV networks are no longer passive on account of the growing presence of DG connected to the LV grid. 

However, bad data in the shape of incorrect measurements and/or outliers can have a significant impact on the state estimation. Early detection of bad data in monitoring devices in secondary substations, i.e., at a decentralized level, is, therefore of enormous importance. This paper has proposed a novel approach to detect bad data in the electronic instrumental transducers (EITs) with the aim of enhancing PSSE. The strength of the contribution made in this work lies in using a highly accurate STLF strategy based on SSA and ANNs.

Finally, the strategy can be extended to other exogenous variables provided that there is data available, such as electricity price, consumer types and other weather factors, e.g cloud cover. Further research could also be done on applying this strategy to solar power forecasting with different forecasting granularity. 

## Figures and Tables

**Table 1:** Error indicators for all prediction models used in this work.

Model	MAPE (%)	MAE (kW)	NRMSE (%)	RMSE (kW)
Weighted average	2.3341	34.5086	3.0682	46.2174
Model 1 (SSA-ANN)	3.1879	46.5177	4.0850	60.3397
Model 2 (ANN)	3.0596	46.1990	4.0892	63.5167
SARIMA prediction	3.3283.	48.1560	4.1985	60.6915
SSA prediction	5.9462	83.9145	7.7610	107.4200

**Table 2:** Scenarios for the error detection.

	δ>4%	δ≤4%
$\;\sqrt{\epsilon_{\alpha }{}^{2}+\epsilon_{\beta }{}^{2}\;}>4\%$	TP	FN
$\;\sqrt{\epsilon_{\alpha }{}^{2}+\epsilon_{\beta }{}^{2}\;}\leq 4\%$	FP	TN

**Table 3:** Normalized error $\left(\epsilon_{\mathrm{norm}}\right)$ as a function of the injected error for a random week.

$\;\epsilon_{norm}\;$		$\;\epsilon_{\beta }\;$										
		−5%	−4%	−3%	−2%	−1%	0%	1%	2%	3%	4%	5%
$\epsilon_{\alpha }$	−5%	0.071	0.078	0.086	0.111	0.112	0.120	0.138	0.152	0.178	0.162	0.147
	−4%	0.094	0.106	0.120	0.134	0.146	0.150	0.171	0.205	0.207	0.186	0.164
	−3%	0.103	0.120	0.141	0.167	0.190	0.201	0.223	0.258	0.247	0.210	0.180
	−2%	0.112	0.135	0.167	0.212	0.269	0.301	0.316	0.328	0.291	0.256	0.213
	−1%	0.118	0.146	0.190	0.269	0.423	0.602	0.499	0.415	0.362	0.278	0.225
	0%	0.120	0.150	0.201	0.301	0.602	∞	0.713	0.464	0.382	0.286	0.229
	1%	0.118	0.146	0.190	0.269	0.423	0.702	0.574	0.415	0.362	0.281	0.227
	2%	0.130	0.157	0.195	0.248	0.314	0.353	0.363	0.363	0.318	0.280	0.233
	3%	0.120	0.140	0.165	0.195	0.222	0.235	0.257	0.284	0.273	0.251	0.215
	4%	0.110	0.125	0.141.	0.158.	0.171.	0.177	0.197.	0.232	0.251	0.222	0.196
	5%	0.100	0.110	0.121	0.131	0.138	0.141	0.159	0.193	0.215	0.198	0.179
